# Timeliness of Clinic Attendance Is a Good Predictor of Virological Response and Resistance to Antiretroviral Drugs in HIV-Infected Patients

**DOI:** 10.1371/journal.pone.0049091

**Published:** 2012-11-07

**Authors:** Mathieu Bastard, Loretxu Pinoges, Suna Balkan, Elisabeth Szumilin, Cecilia Ferreyra, Mar Pujades-Rodriguez

**Affiliations:** 1 Epicentre, Paris, France; 2 Médecins Sans Frontières, Paris, France; 3 Médecins Sans Frontières, Barcelona, Spain; Boston University, United States of America

## Abstract

**Background:**

Ensuring long-term adherence to therapy is essential for the success of HIV treatment. As access to viral load monitoring and genotyping is poor in resource-limited settings, a simple tool to monitor adherence is needed. We assessed the relationship between an indicator based on timeliness of clinic attendance and virological response and HIV drug resistance.

**Methods:**

Data from 7 virological cross-sectional studies were pooled. An adherence indicator was calculated as the number of appointments attended with delay divided by the number of months between antiretroviral treatment (ART) initiation and date of virological testing and multiplying this by 100. Delays of 1 or more to 5 or more days were considered in turn. Multivariate random-intercept logistic regression was fitted to examine the effect on outcomes, separately for adults and children.

**Results:**

A total of 3580 adults and 253 children were included. Adults were followed for a median of 26.0 months (IQR 12.8-45.0) and attended a median of 24 visits (IQR 13–34). The 1-day delay adherence indicator was strongly associated with viral load suppression (OR 0.96, 95% CI 0.95–0.97 per unit increase), virological failure (OR 1.05, 95% CI 1.03–1.06) and HIV drug resistance (OR 1.03, 95% CI 1.01–1.05) after adjusting for initial age and CD4 count, previous ART experience, type of regimen and Tuberculosis diagnosis at start of therapy. Similar results were observed in children.

**Conclusion:**

An adherence indicator based on timeliness of clinic attendance predicts strongly both virological response and drug resistance, and could help to timely identify non-adherent patients in settings where viral load monitoring is not available.

## Introduction

Since the introduction of combined active antiretroviral therapy (ART), mortality and morbidity due to HIV have been drastically reduced in high-, middle- and low-income countries [1], [2]. ART is a life-long treatment and the success of therapy is strongly related to patients achieving and maintaining good levels of adherence to antiretroviral regimens [3]–[7]. It is therefore essential to monitor patient adherence to ART, so as to timely identify poorly adherent patients, and to develop targeted interventions for optimising and maintaining adherence levels over time.

Viral load monitoring and genotyping are frequently used to identify non-adherent patients. However, access to these laboratory diagnostics is often poor in resource-limited settings. Simple alternative tools for rapidly detecting patients who do not regularly take their antiretroviral drugs as prescribed are therefore urgently needed [8], [9].

No gold standard method exists for assessing treatment adherence. Many tools have been proposed, including pill-count, self-reported adherence, various biological markers, attendance to drug refill visits and/or clinic attendance, and quantitative determination of drugs in blood [10], [11]. Each of these methods has advantages and drawbacks and some cannot be implemented routinely by HIV programmes in resource-limited countries [10], [11]. Furthermore, adherence is influenced by many patient-, time- and treatment-related factors (which also interact with one another), increasing the complexity of any assessment [12]–[14].

To monitor the emergence of drug resistance in patients receiving ART, the World Health Organization (WHO) recommends that HIV programmes use a number of indicators, called “early warning indicators” [15]. One of these is patients’ on-time appointment-keeping, an indicator we hypothesised might also correlate with the level of ART adherence and therefore be useful in predicting adherence behaviour within a patient population.

To assess whether a simple tool based on regularity of patients’ follow-up clinic attendance can be used as a proxy for treatment adherence, we pooled data from cross-sectional virological studies conducted in HIV programmes supported by Médecins Sans Frontières (MSF), linked them back to longitudinal monitoring data and examined its relationship with viral load suppression, virological failure and resistance to antiretroviral drugs.

## Methods

### Study Design

**Figure 1:**
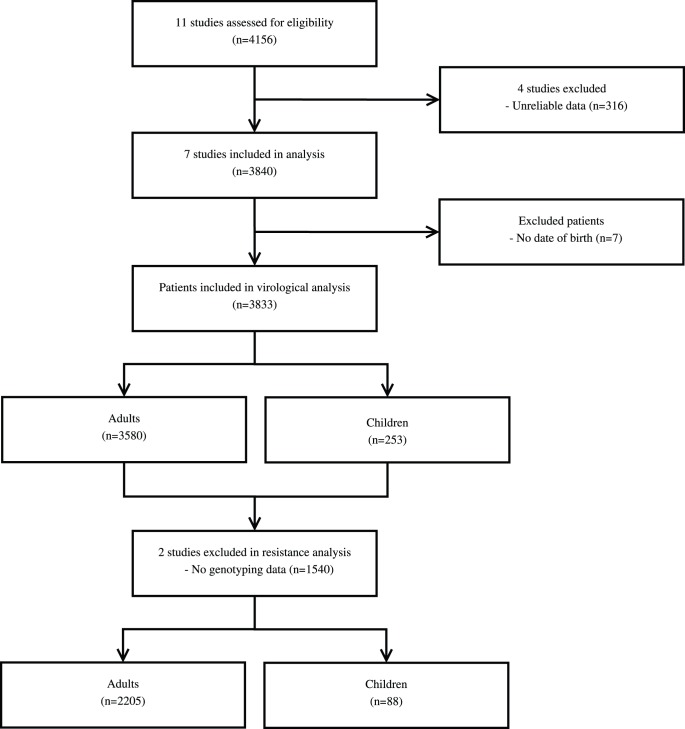
Flow chart of the patients included in the study.

This is a cross-sectional secondary data analysis of cross-sectional virological evaluations conducted among patients receiving ART for 6 months or more in MSF-supported HIV programmes. Data were linked back to individual patient monitoring data collected in the HIV programs. Of 11 cross-sectional studies conducted between 2004 and 2009 in Africa and Asia, 4 were excluded because virological information was either unreliable or missing (studies in Zambia, Burkina Faso, Kenya and Cameroon). Immunovirological testing was done in all studies and genotype testing in 5 studies (Figure 1). In two studies plasma HIV RNA quantification was performed using the Cobas Amplicor HIV-1 Monitor v1.5 reverse transcriptase PCR (Roche Diagnostics, Meylan, France), which targets the gag p24 region of the virus [16], [17]. In the three other studies, the Agence Nationale de Recherche sur le SIDA et les Hépatites Virales (ANRS) generic real-time PCR test was used [18]–[20]. The detection threshold for viral load determination varied from 40 to 400 copies/mL across studies. Genotyping methods used to identify drug resistance were the dideoxy chain termination method (ABI PRISM Ready Reaction AmpliTaq Fs; Dye Deoxy Terminators; Applied Biosystems, Paris, France) in two studies [16], [17] and the standard methods developed by the ANRS Resistance study group in 3 studies [18]–[20]. The threshold for genotype testing ranged from 250 to 5000 copies/mL [16]–[20]. Recommendations were made to the Epicentre collaborating laboratories to submit the sequence files from study patients to GenBank.

Routine clinical and laboratory data, such as appointment dates and the dates of clinic visits, were collected prospectively in each of the sites using the FUCHIA software (Follow-Up and Care of HIV Infection and AIDS, Epicentre, Paris). Viral load testing was not routinely performed in these programs and it was only used when treatment failure was suspected.

### Definitions

In the analyses, viral load suppression was defined as <400 copies/mL and virological failure as >5000 copies/mL (as defined in current WHO guidelines [21]). Resistance was defined as the presence of one or more non-nucleoside reverse transcriptase inhibitor (NNRTI) or nucleoside reverse transcriptase inhibitor (NRTI) high-level resistance mutations to antiretroviral drugs, using the Stanford University’s genotypic resistance database and test result interpretation algorithm (http://hivdb.stanford.edu/) [22], [23].

In MSF-supported HIV programs, drug refills including provision of buffer stocks of 1–3 months, usually take place during clinic visits but antiretroviral drugs are not provided in all medical visits. In the present study, we used a proxy indicator for patient adherence to therapy based on the timeliness of patient attendance to clinic visits [24]. Briefly, we divided the number of appointments attended with delay by the number of months of follow-up between the date of ART initiation and the date of virological testing and multiplied this by 100. Adherence was considered good if patients were delayed less than 5% of the time in attending their appointments, moderate if they were delayed attending between 5% and 19% of their appointments, and poor if they were delayed attending 20% or more of their appointments.

### Statistical Analysis

Data were analysed separately for adult (≥15 years) and paediatric (<15 years) patients. Patient characteristics, numbers of visits, and intervals between consecutive visits during the study follow-up were summarised using standard descriptive statistics. Comparison of visits across studies was assessed using Kruskal-Wallis tests.

Five separate continuous adherence indicators were calculated for each patient, as explained before, using 1 or more, to 5 or more days of delay in clinic attendance in turn. Using univariate random-intercept logistic regression and Wald statistics, we selected the delay (in number of days) for which the continuous adherence indicator was most strongly associated with viral load suppression, virological failure and HIV drug resistance and used this in subsequent analyses.

Univariate and multivariate random-intercept logistic regression models were fitted to assess the association between the continuous and categorical adherence indicators and viral load suppression, virological failure and HIV drug resistance after adjustment for other confounding factors at baseline: sex, age, CD4 cell count (<50, 50–199, ≥200/µL and unknown for adults; <200 and ≥200/µL for children), WHO clinical stage (1 or 2, 3, 4 and unknown), body mass index (BMI), tuberculosis diagnosis, history of ART use and type of regimen (nevirapine and efavirenz for non-nucleoside reverse transcriptase inhibitor (NNRTI), zidovudine and stavudine for nucleoside reverse transcriptase inhibitor (NRTI)) at ART initiation.

Two sensitive analyses were performed. First, we did separate analyses for patients who received ART for <24 months and for ≥24 months. We also repeated the analyses using only data from the subgroup of patients with complete CD4 cell count, clinical stage and BMI information.

Statistical analyses were performed with Stata 11.2 software (Stata Corporation, College Station, Texas, USA).

### Ethical Review

Before conducting the cross-sectional studies included in the analysis, ethics committee approval had been obtained. The ethics committees for the different studies were: the Uganda National Council for Science and Technology, the Ugandan AIDS Research Committee, the National Ethics Committee of Cambodia, KEMRI National Ethical Review Committee of Nairobi (Kenya), the National Health Science Research Committee of Malawi and the Consultative Committee for People involved in Biochemical Research (CPPRB) of Saint Germain-en-Laye, Paris, France. All participating patients had provided written informed consent [16]–[20].

## Results

A total of 3840 patients participated in the 7 cross-sectional studies included in our analysis. Seven patients (0.2%) were excluded because their date of birth was missing. Of the remaining 3833 patients, 3580 were adults and 253 children.

Patient characteristics at ART initiation are shown in Table 1. Most patients were treated in Kenyan (39.8%) and Malawian (35.4%) HIV programmes. Median age for adults was 36.0 years (interquartile range (IQR) 30.5–42.7) and for children 5.1 years (IQR 2.7–8.2). About two thirds of adults and half of children were females; 97% of patients had no history of ART use. Initial median CD4 cell counts were 119 cells/µL (IQR 55–182) and 286 (IQR 178–608), respectively. Fifty-three percent of adults were in clinical stage 3 and 29.3% in stage 4, median BMI was 19.9 kg/m^2^ (IQR 18.2–21.8) and 305 patients (8.5%) were diagnosed with tuberculosis. Among children, 51% were in clinical stage 3 and 17.4% in stage 4, median BMI was 14.9 kg/m^2^ (IQR 13.8–16.2) and 7 patients (2.8%) were diagnosed with tuberculosis. The most commonly prescribed initial ART regimens were a combination of stavudine, lamivudine and either nevirapine (83.7% for adults and 91.1% for children) or efavirenz (11.9% and 8.5%, respectively).

**Table 1:** Patient characteristics at ART start by age.

Characteristics	Adults (N = 3580)	Children (N = 253)
**Site (%)**		
Study 1, Malawi	1267 (35.4)	81 (32.0)
Study 2, Kenya	924 (25.8)	2 (0.8)
Study 3, Uganda	506 (14.1)	86 (34.0)
Study 4, Kenya	502 (14.0)	84 (33.2)
Study 5, Cambodia	381 (10.7)	0 (0.0)
**Age, years**		
Median [IQR]	36.0 [30.5–42.7]	5.1 [2.7–8.2]
**Sex (%)**		
Men	1293 (36.1)	125 (49.5)
Women	2287 (63.9)	128 (50.5)
**History of ART (%)**		
Yes	108 (3.0)	4 (1.6)
No	3472 (97.0)	249 (98.4)
**CD4 cell count, cells/µL**		
Median [IQR]	119 [55–182]	286 [178–608]
<50	659 (18.4)	16 (6.3)
50–199	1700 (47.5)	47 (18.6)
≥200	509 (14.2)	136 (53.8)
Unknown	712 (19.9)	54 (21.3)
**Clinical stage (%)**		
1 or 2	606 (16.9)	64 (25.3)
3	1885 (52.7)	129 (51.0)
4	1047 (29.3)	44 (17.4)
Unknown	42 (1.1)	16 (6.3)
**BMI, kg/m^2^**		
Median [IQR]	19.9 [18.2–21.8]	14.9 [13.8–16.2]
<18.5	1040 (29.0)	99 (39.1)
≥18.5	2502 (69.9)	134 (53.0)
Unknown	38 (1.1)	20 (7.9)
**Tuberculosis diagnosis (%)**		
No	3275 (91.5)	246 (97.2)
Yes	305 (8.5)	7 (2.8)

Note: BMI, body mass index, categorized for children according to Cole *et al*. classification [32].

**Figure 2:**
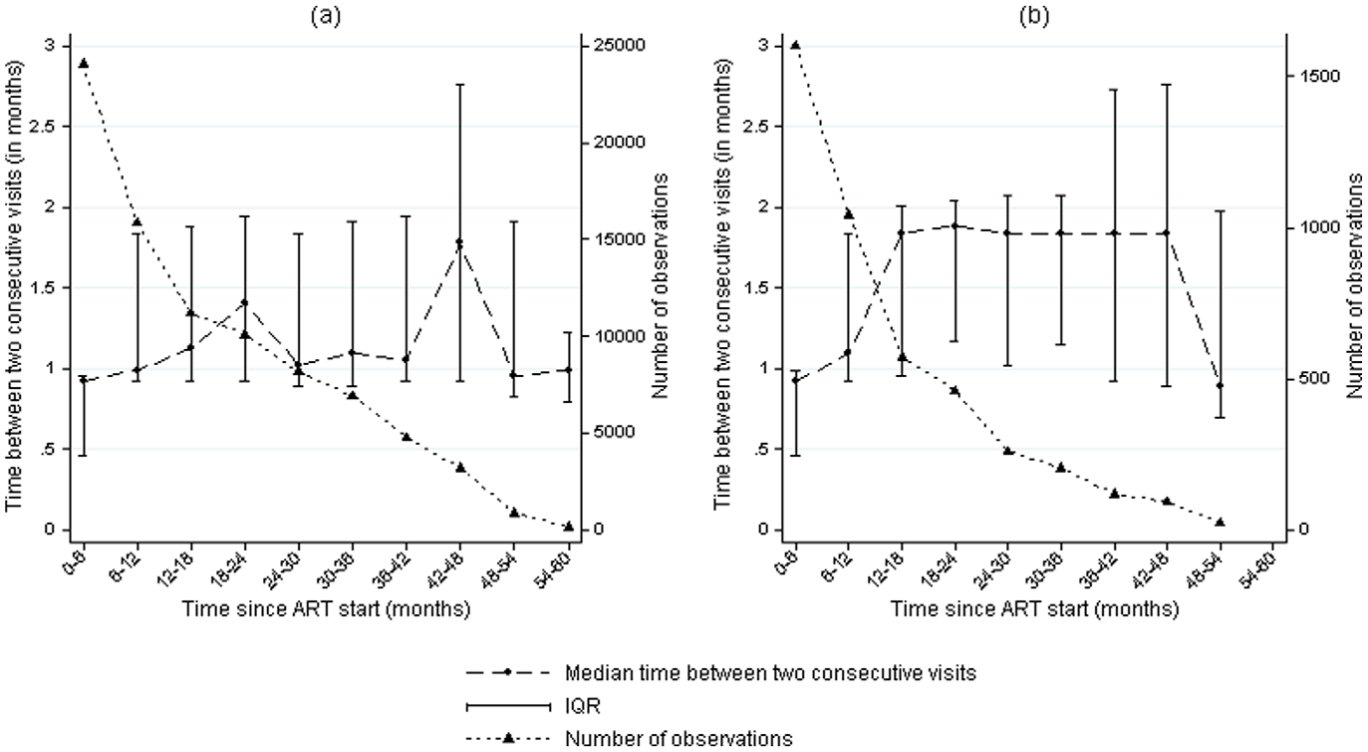
Variation in the time between two consecutive visits with length of follow-up since ART initiation in adults (a) and children (b).

Median follow-up time from ART initiation was 26.0 months (IQR 12.8–45.0) for adults and 22.6 months (IQR 11.1–28.1) for children. The median numbers of visits per patient were 24 (IQR 13–34) and 17 (IQR 11–24), respectively. Median intervals between consecutive visits were 0.95 months (IQR 0.82–1.84) and 1.02 months (IQR 0.89–1.84), respectively, and remained stable over time on ART (Figure 2).

At the time of the cross-sectional evaluation, 3021 out of 3580 adult patients (84.5%) were virologically suppressed while 248 (6.9%) had virological failure. Genotype testing was performed for 2205 adult patients who had a viral load >1000 copies/mL. A total of 123 (5.6%) patients were classified as having HIV drug resistance to at least one antiretroviral drug. Similarly, 144 out of 253 pediatric patients (56.9%) failed to suppress viral load while 79 (31.2%) had virological failure. Genotype testing was performed for 33 children who had a viral load >1000 copies/mL. A total of 29 (11.5%) children had HIV drug resistance to at least one antiretroviral drug. The frequency of profiles of NRTI and NNRTI mutations encountered is shown in Tables S1 and S2.

The use of the continuous adherence indicator based on a delay of 1 or more days after the scheduled appointment date showed the strongest association with viral load suppression, virological failure and HIV drug resistance in univariate analyses (Wald statistics of –6.50, 6.32 and 3.13, respectively; data not shown) and was therefore used in the subsequent risk factor analysis.

The overall proportion of appointments attended with delay was 6.38% (95% CI: 6.22–6.55%) for adults and 12.08% (95% CI: 11.13–13.07%) for children, and decreased slightly following ART initiation. The median numbers of appointments attended with delay per patient were 1 (IQR 0–2) and 2 (IQR 0–3), respectively. These represent medians of 4.59% (IQR 0–11.11) and 9.09% (IQR 0–20.0%) of appointments attended with delay per patient, respectively. Median numbers and proportions of appointments attended with delay differed across studies (*P*<0.001).

### Association between Adherence and Virological Outcomes in Adults

**Figure 3:**
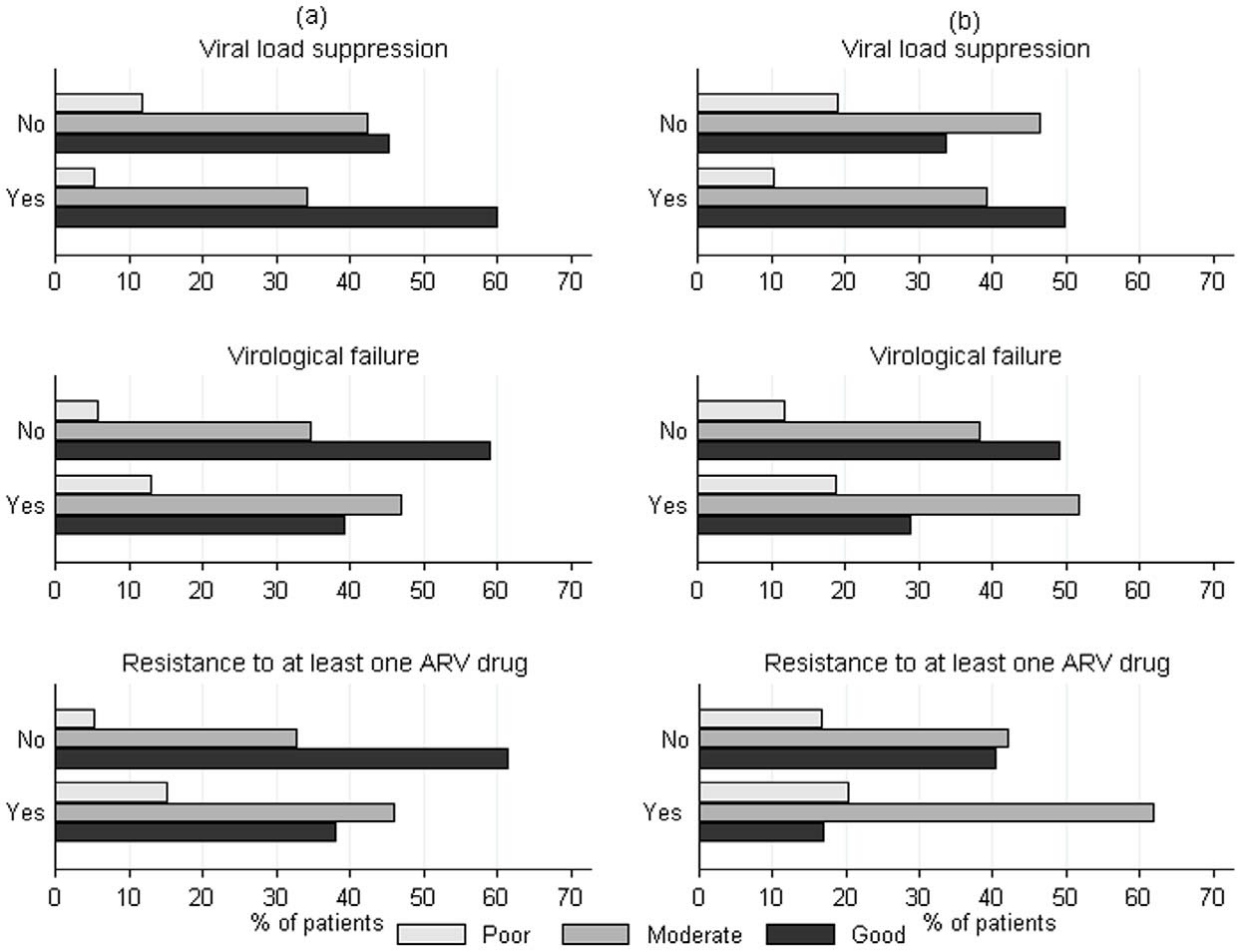
Patient distribution of adherence to clinic visits by outcome in adults (a) and children (b).

The median value of the continuous adherence indicator in the full dataset was 4.08% (IQR 0–9.06%) and ranged from 0% (IQR 0%–2.01%) to 7.93% (IQR 0%–9.91%) across studies. According to the study classification, 58.0% of patients showed good adherence, 35.6% moderate and 6.4% poor. The categorisation of patients according to adherence levels (categorical indicator) showed higher proportions of individuals with poor and moderate adherence among those with virological failure and HIV drug resistance, and higher proportions of individuals with good adherence among those with viral load suppression (Figure 3).

In univariate analysis using the continuous adherence indicator, patients with higher adherence indicator values (e.g. higher incidence of appointments attended with delay) were less likely to achieve viral load suppression (Odds Ratio (OR) 0.96, 95% CI 0.95–0.97 per unit increase), and more likely to experience virological failure (OR 1.05, 95% CI 1.03–1.06) and HIV drug resistance (OR 1.03, 95% CI 1.01–1.05).

In multivariate analyses (Table 2), the categorical adherence indicator was strongly associated with viral load suppression (OR 0.70, 95% CI 0.57–0.86 for moderate adherence, and OR 0.35, 95% CI 0.25–0.49 for poor adherence, compared to good adherence), virological failure (OR 1.86, 95% CI 1.38–2.50 and OR 3.16, 95% CI 2.01–4.99, respectively) and drug resistance (OR 1.70, 95% CI 1.07–2.70 and OR 2.54, 95% CI 1.33–4.86, respectively). Results of random intercept testing were significant (*P*<0.001) and variance due to studies represented less than 5% of the total variance, suggesting low heterogeneity between studies.

**Table 2:** Multivariate random-intercept logistic regressions of experiencing viral load suppression, virological failure and HIV drug resistance for adults and children.

Factors	Viral suppression	Virological failure	HIV drug resistance
	OR (95% CI)	OR (95% CI)	OR (95% CI)
**Adults**			
**Adherence indicator**			
Good	1	1	1
Intermediate	0.70 (0.57–0.86)	1.86 (1.38–2.50)	1.70 (1.07–2.70)
Poor	0.35 (0.25–0.49)	3.16 (2.01–4.99)	2.54 (1.33–4.86)
**Age, years**	1.02 (1.01–1.03)	0.96 (0.95–0.98)	0.95 (0.93–0.98)
**ART experience**			
Yes	1	1	1
No	2.22 (1.34–3.68)	0.46 (0.24–0.91)	0.18 (0.08–0.38)
**CD4 cell count, cells/µL**			
<50	1	1	1
50–199	1.06 (0.80–1.39)†	0.76 (0.53–1.08)	0.55 (0.34–0.87)
≥200	1.08 (0.75–1.55)†	0.58 (0.37–0.98)	0.66 (0.32–1.39)
Unknown	0.83 (0.60–1.15)†	0.99 (0.66–1.52)	0.89 (0.47–1.67)
**NNRTI component of regimen**			
EFV	1	1	1
NVP	1.61 (1.00–2.60)	0.94 (0.53–1.67)†	0.96 (0.39–2.35)†
**Tuberculosis diagnosis**			
No	1	1	1
Yes	1.09 (0.73–1.63)†	0.94 (0.56–1.98)†	1.86 (0.98–3.54)
**Children**			
**Adherence indicator** *			
Good	1	1	1
Intermediate	0.59 (0.34–1.02)	2.22 (1.20–4.11)	3.14 (1.03–9.54)
Poor	0.35 (0.16–0.77)	2.87 (1.25–6.61)	-
**Sex**			
Men	1	1	1
Women	1.83 (1.09–3.06)	0.37 (0.21–0.65)	0.74 (0.28–1.95)†
**CD4 cell count, cells/µL**			
<200	1	1	1
≥200	0.80 (0.43–1.48)†	0.92 (0.48–1.79)†	0.45 (0.17–1.19)†
Unknown	1.02 (0.47–2.20)†	0.71 (0.31–1.65)†	1.14 (0.19–6.90)†

Note: NNRTI, non-nucleoside reverse transcriptase inhibitor; NRTI, nucleoside reverse transcriptase inhibitor.

* Moderate and poor adherence categories were combined in the analysis of HIV drug resistance among children.

† Non-significant factor in the final model.

### Association between Adherence and Virological Outcomes in Children

In the full dataset, the median value of the continuous adherence indicator among children was 7.90% (IQR 0–16.65%). Overall, 43.0% were classified as showing good adherence, 42.7% as moderately adherent and 14.2% as poorly adherent. In multivariate analyses, the categorical adherence indicator remained strongly associated with viral load suppression (OR 0.59, 95% CI 0.34–1.02 for moderate adherence, and OR 0.35, 95% CI 0.16–0.77 for poor adherence, compared to good adherence) and with virological failure (OR 2.22, 95% CI 1.20–4.11 and OR 2.87, 95% CI 1.25–6.61, respectively). A borderline statistical association was observed for drug resistance despite of the small sample size (OR 3.14, 95% CI 1.03–9.54, p = 0.04; Table 2).

### Sensitivity Analyses

Sensitivity analyses showed that the adherence indicator evaluated remained strongly associated with viral load suppression, virological failure and drug resistance regardless of the duration of ART in both adults and children. Furthermore, the estimated effect was similar regardless of the duration of ART (<24 or ≥24 months), and analyses restricted to patients with complete data showed consistent results (data not shown).

## Discussion

In this large multicentric cross-sectional study including 3833 patients, we observed a strong association between an ART adherence indicator based on the regularity of attendance to scheduled clinic appointments and viral load suppression, virological failure and HIV drug resistance. Patients classified as having lower levels of adherence as defined by our indicator were less likely to have undetectable HIV viral load and were two to three times more likely to be in virological failure and have developed resistance mutations to antiretroviral drugs. These associations were seen in both adults and children and did not vary with duration of ART use.

In our study population virological and drug resistance outcomes were more strongly associated with the adherence indicator based on a delay of one or more days after the scheduled clinical visit was used in the calculation. This delay seems reasonable in the context of HIV treatment programmes in resource-limited settings. In our study 6% of patient appointments were attended with a delay of one or more days. Although previous studies have suggested that adherence might decline with time on ART [13], in our analysis, both the proportion of appointments attended with delay and the interval between successive clinic visits remained almost constant over time since treatment initiation. The median treatment duration of study patients was 26 months and 75% of individuals had received therapy for less than 45 months. These findings seem to support the validity of using the proposed adherence indicator to monitor adherence in HIV programmes during the first 3–4 years of ART.

Overall, approximately 57% of patients showed good adherence when the indicator evaluated was used. This figure is lower than estimates reported in previous studies conducted in resource-limited settings, but those studies primarily used self-reported measures of adherence and a 95% cut-off to define good adherence [3], [25]. Lower estimates of effect have been reported in studies where objective measurements of adherence, such as a Medication Event Monitoring System (MEMS), were used [25]. Such objective measurement methods, including delays in clinic attendance, might discriminate better among patients with different levels of adherence and could help to identify higher numbers of non-adherent patients.

The data used for this evaluation included patients from several African and one Asian HIV programme. At the time of the cross-sectional evaluations about 85% of study patients were virologically suppressed, 7% had virological failure and 6% had resistant mutations to antiretroviral drugs. Despite the relatively low rates of failure and drug resistance, a high and statistically significant increase in the probability of these two outcomes was observed in patients classified by the proposed indicator as moderate or low adherent, compared with patients classified as good adherent. This effect was independent of age, previous exposure to ART or the CD4 cell count level at ART initiation, and of the type of antiretroviral regimen taken. Although the absence of viral load monitoring in the study sites did not allow us to distinguish between episodes of long- versus short-term viremia in the analyses, our findings are strengthened by the consistent results of an association between the three virological outcomes studied and the adherence indicator (considered as either continuous or categorical variable). These outcomes are key markers of true adherence and treatment success.

The association between the virological outcomes and the adherence indicator was also strong in children despite the smaller sample size of this group of patients, and it was of borderline significance for antiretroviral resistance, probably due to lack of power. This is an important finding that supports the use of this same indicator to identify non-adherent adult and children patients.

We previously described strong associations between the adherence indicator evaluated, failure to first- and second-line therapy (primarily defined by WHO clinico-immunological criteria), and mortality among patients using first- and second-line therapy [26], [27]. Several studies have reported an association between virological outcomes and attendance to drug-refill visits and/or clinic attendance [28]–[31]. However, these studies were generally smaller in size and/or conducted in a single centre, limiting the generalisability of the findings. Strengths of our study are its large sample size, the inclusion of patients coming from seven different HIV treatment programmes in sub-Saharan Africa and Southeast Asia, the availability of genotyping drug resistance data to confirm the results of the evaluation, and the consistent results found in children and adult patients. The multivariate mixed model indicated low heterogeneity between the centres included in the evaluation and supports the use of the indicator in other sub-Saharan African and Southeast Asian HIV programmes.

Our study has several limitations. First, patient follow-up on ART differed greatly according to site, from about 6 to 60 months, depending on the eligibility criteria used in each cross-sectional evaluation. As a result, the number of clinic visits per patient varied widely across sites, which could affect the reliability of the adherence indicator. To assess whether the duration of follow-up affects our findings, we performed a sensitivity analysis to identify possible differences in the degree of association between patients followed for less than 24 months and those followed for more than 24 months. No evidence of differences was detected for viral suppression, virological failure or HIV drug resistance. In addition, the length of delay in clinic attendance was not considered in the calculation of the indicator. Nevertheless, the median duration of clinic delay was 3 days and only 3.2% of visits had delays of more than 5 days.

Our indicator might tend to underestimate patient adherence, since patients coming for more than one day after their scheduled visit are considered non-adherent for the entire period between the last visit and the actual visit, which is probably not the case (buffer drugs given to patients could prevent treatment interruptions). Because we calculated the adherence indicator over the whole period of ART use, we could not assess the temporal relationship between exposure and outcome and the effect of time itself was not really taken into account. Nevertheless, the homogeneous distribution of appointments attended with delay over time gives further confidence in the indicator. For the same reason, another important finding is that the interval between successive visits (on average about 1 month) was almost constant over time on ART.

In conclusion, in this multicentric study conducted in resource-limited settings we observed a strong association between an indicator of adherence based on regularity of clinic attendance and both virological response and drug resistance. This indicator represents a simple tool that could be used in HIV treatment programmes for prompt identification of non-adherent patients who need strong, targeted support and counselling to prevent virological failure and drug resistance development.

## Supporting Information

Table S1 **Number of adult patients with specific profiles of NRTI and NNRTI mutations stratified by amount of viral load.**
(DOC)

Table S2 **Number of paediatric patients with specific profiles of NRTI and NNRTI mutations stratified by amount of viral load.**
(DOC)
