# Supplementary material for: Timeliness of Clinic Attendance Is a Good Predictor of Virological Response and Resistance to Antiretroviral Drugs in HIV-Infected Patients
Source: PLoS One. 2012 Nov 7;7(11):e49091. doi: 10.1371/journal.pone.0049091 (PMC3492309; doi:10.1371/journal.pone.0049091)
Supplement: Table S1 — Number of adult patients with specific profiles of NRTI and NNRTI mutations stratified by amount of viral load. (DOC) [file pone.0049091.s001.doc]

**Table S1. Number of adult patients with specific profiles of NRTI and NNRTI mutations stratified by amount of viral load**

| **NRTI mutations** | **NNRTI mutations** | **No. of patients** |
| --- | --- | --- |
| **Viral load 1000-5000 copies/mL** |  |  |
| - | V179I | 8 |
| M184V | K103N | 4 |
|  | K103N | 2 |
| M184V | K101E G190A | 2 |
| M184V | Y181C | 2 |
| - | V179I/V | 1 |
| - | K103T/K | 1 |
| - | V179I/V Y188C/Y | 1 |
| - | V179I G190E/G | 1 |
| D67D/G M184V | K103N | 1 |
| K65R | K103N Y181C G190A/G | 1 |
| K65R | V106A Y181C | 1 |
| K65R M184V | K103N | 1 |
| K65R M184V | V106M Y181C | 1 |
| K65R T69DEL | Y181C G190A | 1 |
| K65R V118I | Y181C Y188H | 1 |
| K65R Y115F | K101E/K V106M G190A | 1 |
| M184V | V106A M230K | 1 |
| M184V | V179I G190A M230P | 1 |
| M184V | K103N Y181C | 1 |
| M184V L210F | Y181C | 1 |
| M41L/M V75I M184V T215Y | K103N Y181C/Y | 1 |
| T69A/T K70R/K M184V K219E/K | K103N Y181C G190A/G | 1 |
| V75I/V M184V | K101E/Q V179I G190A | 1 |
| **Viral load >5000 copies/mL** |  |  |
| M184V | K103N | 7 |
| M184V | Y181C | 5 |
| M184V | K101E G190A | 4 |
| - | V179I | 3 |
| - | K103N | 2 |
| - | Y181C | 2 |
| D67N L210W M41L M184V T215Y | V179I Y181V | 2 |
| M184V | K103N P225H | 2 |
| M184V | K103 | 2 |
| M184V | K103N V179I | 2 |
| - | V106M | 1 |
| - | G190S | 1 |
| D67N K70R M184V K219 | K103 V179 P225H | 1 |
| D67N K70R M184V K219E | Y181C | 1 |
| D67N K70R/K M184V | Y181C | 1 |
| D67N M41L M184V Q151M T215F | K103N | 1 |
| D67N T69N Y115F Q151M M184V | K103N | 1 |
| D67N/D K70R/K M184V | K101E K103N | 1 |
| F116Y/F Q151L/M | - | 1 |
| K65R | Y181S Y188H | 1 |
| K65R T69A V75I F116Y Q151M M184V | K103N V179S Y181C | 1 |
| K65R/K T69N/T V75T M184V | K103N Y181C | 1 |
| K70R M184V | V108I Y181C | 1 |
| K70R M184V T215I K219E | L100I K103N | 1 |
| K70R M184V T69N | K103N L100I | 1 |
| K70R/K M184V | K103N V179I | 1 |
| K70R/K M184V T215F | K103N V106I/V V179I | 1 |
| L210S M41L M184V T215Y | Y188L | 1 |
| L210W M41L M184V T215Y | V108I V179I Y181C | 1 |
| L74V/L M184V | V179I Y181C | 1 |
| M184I/M/V | Y181C | 1 |
| M184I/M/V | V108I/V Y181C | 1 |
| M184V | K101R V179I Y181C/Y | 1 |
| M184V | K103N V179I/V | 1 |
| M184V | K103N/K V179I Y181C | 1 |
| M184V | K101H V179I Y181C G190A | 1 |
| M184V | K101E | 1 |
| M184V | Y188L | 1 |
| M184V | K101E K103N/K G190S | 1 |
| M184V | K103N/K | 1 |
| M184V | V179I G190A | 1 |
| M184V | G190A K101A V179I | 1 |
| M184V | K103N V179I Y181C | 1 |
| M184V | V108I/V Y181C | 1 |
| M184V | K101E Y181C/Y G190A | 1 |
| M184V | K103N G190A | 1 |
| M184V | K103N G190A/G | 1 |
| M184V | K103N V108I/V | 1 |
| M184V | K103S V179I | 1 |
| M184V | K103N/S V106M | 1 |
| M184V | K103N Y188H/Y | 1 |
| M184V T215F | K101E G190A | 1 |
| M184V T215Y | K101E V179I G190A | 1 |
| M184V T215Y | K103N | 1 |
| M184V T215Y | K103N V108I | 1 |
| M184V T215Y | G190A K101E | 1 |
| M41L D67N T69D L74V M184V L210W T215Y | K103N M230L | 1 |
| M41L K70R M184V K219E | V108I/V Y181C | 1 |
| M41L M184V T215F | G190A V179I | 1 |
| M41L M184V T215Y | V108I V179I Y181C | 1 |
| M41L M184V T215Y | K103N | 1 |
| M41L M184V T215Y | A98G K103N | 1 |
| M41L M184V T215Y V75I | V179I Y181C | 1 |
| M41L M184V T215Y V75I | K103N V90I V179I | 1 |
| M41L V75I M184V T215F | L100I/L K101Q V106M | 1 |
| M41L/M D67N/D M184V L210W T215Y | Y181I | 1 |
| M41L/M V75I M184V | K103N | 1 |
| M41V M184V | K103N Y181C | 1 |
| T69N M184V T215Y | Y181C | 1 |
| T69N/T V75I M184V T215Y | K103N Y181C/Y | 1 |
| T69P/T M184V | V179I Y181C | 1 |
| T69S M184V | K103N V108I V179I | 1 |
| V118I M184V | Y181C | 1 |
| V75I M184V | K103N V179I | 1 |
| V75I M184V | Y181C | 1 |
| V75M M184V L210W/L T215F | V106A | 1 |
| V75I/V M184V | K101E/Q V179I G190A | 1 |
