# Supplementary material for: Timeliness of Clinic Attendance Is a Good Predictor of Virological Response and Resistance to Antiretroviral Drugs in HIV-Infected Patients
Source: PLoS One. 2012 Nov 7;7(11):e49091. doi: 10.1371/journal.pone.0049091 (PMC3492309; doi:10.1371/journal.pone.0049091)
Supplement: Table S2 — Number of paediatric patients with specific profiles of NRTI and NNRTI mutations stratified by amount of viral load. (DOC) [file pone.0049091.s002.doc]

**Table S2. Number of paediatric patients with specific profiles of NRTI and NNRTI mutations stratified by viral load**

| **NRTI mutations** | **NNRTI mutations** | **No. of patients** |
| --- | --- | --- |
| **Viral load 1000-5000 copies/mL** |  |  |
| - | V179I | 1 |
| M184V | K103N V108I/V V179I | 1 |
| M184V | K101Q/K V108I/V Y181C | 1 |
| M184V | K101E V179I G190A | 1 |
| M184V | K101Q V179I G190A | 1 |
| **Viral load >5000 copies/mL** |  |  |
| M184V | Y181C | 2 |
| M184V | V179I G190A | 2 |
| D67N M184V T215Y | K101Q K103N | 1 |
| D67N T69D K70R M184V T215F K219Q | V179I Y181V | 1 |
| D67N T69D/T K70R M184V T215I/T K219E/Q | Y181C/Y G190A | 1 |
| K65R D67G | Y181C G190S | 1 |
| M184V | K103N | 1 |
| M184V | K103N/K G190A/G | 1 |
| M184V | V179I/V Y188L | 1 |
| M184V | K101E/K Y181C/Y G190A/G | 1 |
| M184V | K101Q V106A | 1 |
| M184V | V179I Y181C G190A | 1 |
| M184V | K101E Y181C/Y G190A | 1 |
| M184V | Y188L | 1 |
| M184V | V179I G190A M230L/M | 1 |
| M184V | K101E Y181C | 1 |
| M184V T215F/T | K103R/K V179E Y181C/Y G190A/G | 1 |
| M184V T215Y | Y181C | 1 |
| M184V T215Y | V106A Y181C | 1 |
| M184V/I T215F/I | Y181C | 1 |
| M41L L74I/L M184V L210W/L T215Y | K103N V179I | 1 |
| M41L T69N/T M184V/M L210W T215Y | V108I Y181C | 1 |
| V75I M184V | V179I Y181C | 1 |
